# Case Report: A rare complication easily misdiagnosed as bacterial peritonitis: a case of sterile peritoneal dialysis-chemical peritonitis induced by amino-acid-based dialysate and literature review

**DOI:** 10.3389/fneph.2026.1917133

**Published:** 2026-09-14

**Authors:** Dongmei Liu, Juan Mou, Hongmei Dai

**Affiliations:** Department of Nephrology and Hematology, Yunyang County People's Hospital, Chongqing, China

**Keywords:** amino-acid-based dialysate, leukocyte counts, mononuclear cells, peritoneal dialysis-chemical peritonitis, renal disease

## Abstract

Peritoneal dialysis-chemical peritonitis induced by amino acid-based peritoneal dialysis solution is extremely rare. There are only a few reported case series of this condition. It can occur at any time during the use of amino acid dialysate, and most cases present with abdominal pain, cloudy effluent, and elevated peritoneal leukocyte counts, closely mimicking infectious peritonitis. This similarity poses a significant diagnostic challenge, as it is difficult to distinguish from bacterial peritonitis. Here, we report a case of peritoneal dialysis-chemical peritonitis associated with amino acid dialysate in a patient on peritoneal dialysis, aiming to raise awareness among clinicians of this rare entity that can be easily confused with infectious peritonitis. We also review its clinical characteristics, differential diagnosis, and management.

## 1 Introduction

For patients with end-stage renal disease (ESRD), the two primary treatment modalities are hemodialysis (HD) and peritoneal dialysis (PD). Relative to HD, PD offers several advantages, including home-based therapy, improved hemodynamic stability, and greater preservation of residual renal function—advantages that have driven its increasing clinical adoption (1). Nevertheless, protein-energy wasting (PEW), characterized by hypoalbuminemia, muscle wasting, and undernutrition, is highly prevalent in PD patients and adversely impacts their prognosis. Daily losses of protein and amino acids via PD, coupled with contributing factors such as reduced appetite and metabolic acidosis, lead to a markedly higher prevalence of PEW in PD patients than in those undergoing HD. Accordingly, PEW has been established as an independent risk factor for mortality in this population (2).

Therefore, correcting PEW is critical in PD management. Amino-acid-based peritoneal dialysis solution containing 1.1% amino acids (e.g., Nutrineal) replaces glucose as the osmotic agent, thereby supplementing amino acids while achieving ultrafiltration and offering dual dialysis–nutrition support. Daily use of one bag of this solution improves serum albumin, prealbumin, and SGA scores in hypoalbuminemic PD patients, with efficacy and ultrafiltration comparable to conventional glucose-based dialysate (2); long-term combined use in pediatric patients also maintains positive nitrogen balance and stable ultrafiltration (3). Collectively, these findings confirm the clinical value of amino-acid-based PD solutions in improving nutritional status and mitigating PEW-related risks.

We report a case of peritoneal dialysis-chemical peritonitis following the inappropriate use of a 1.1% amino acid-containing peritoneal dialysis solution, aiming to raise clinical awareness.

The indication for peritoneal dialysis solution containing 1.1% amino acids is peritoneal dialysis patients with serum albumin levels below 35 g/L. Although amino acid dialysis solutions are generally safe, they may still be associated with adverse reactions. One rare but noteworthy adverse event is aseptic peritoneal dialysis-chemical peritonitis (peritoneal dialysis-associated chemical peritonitis) (3, 4). This event is characterized by the onset of abdominal pain, cloudy dialysate effluent, and elevated peritoneal white blood cell count following the use of amino acid dialysis solution, with negative bacterial and fungal cultures. Symptoms typically resolve spontaneously after discontinuation of the amino acid-containing solution (5). Although most reported cases in the literature are sporadic (3, 5, 6), the clinical presentation of this complication is nearly indistinguishable from infectious peritonitis, potentially leading to unnecessary broad-spectrum antibiotic treatment or even catheter removal (7). Therefore, increasing awareness of this rare but reversible cause is essential for timely recognition and prevention of overtreatment.

## 2 Case report

A 39-year-old male patient was initially diagnosed with chronic renal failure (etiology unspecified) more than five years ago based on an elevated serum creatinine level of 200 μmol/L, and the disease gradually progressed to stage 5 chronic kidney disease (CKD stage 5), corresponding to end-stage renal failure (uremic phase), over the subsequent three years. On November 14, 2022, he underwent manual placement of a coiled peritoneal dialysis catheter under transversus abdominis plane anesthesia. Due to inadequate peritoneal dialysis adequacy, the dialysis regimen was adjusted on November 16, 2025, as follows: 2 L of 1.5% low-calcium peritoneal dialysis solution administered intraperitoneally three times daily, combined with 2L of 2.5% low-calcium peritoneal dialysis solution administered intraperitoneally twice daily (Monday to Saturday), along with hemodialysis performed on Sundays.

On January 8, 2026, the patient noted cloudy peritoneal dialysate effluent (**Figure 1**) in the absence of fever, chills, or abdominal pain. Laboratory evaluation revealed a peripheral white blood cell count of 5.19×10^9^/L, a neutrophil count of 3.96×10^9^/L, and normal C-reactive protein (CRP) and procalcitonin (PCT) levels. Dialysate analysis showed pale yellow, slightly turbid fluid, a Rivalta test (±), and a total nucleated cell count of 543×10^6^/L (84% mononuclear, 16% polymorphonuclear). Cefixime (0.1 g orally twice daily) was initiated as prophylactic anti-infective therapy.

**Figure 1:**
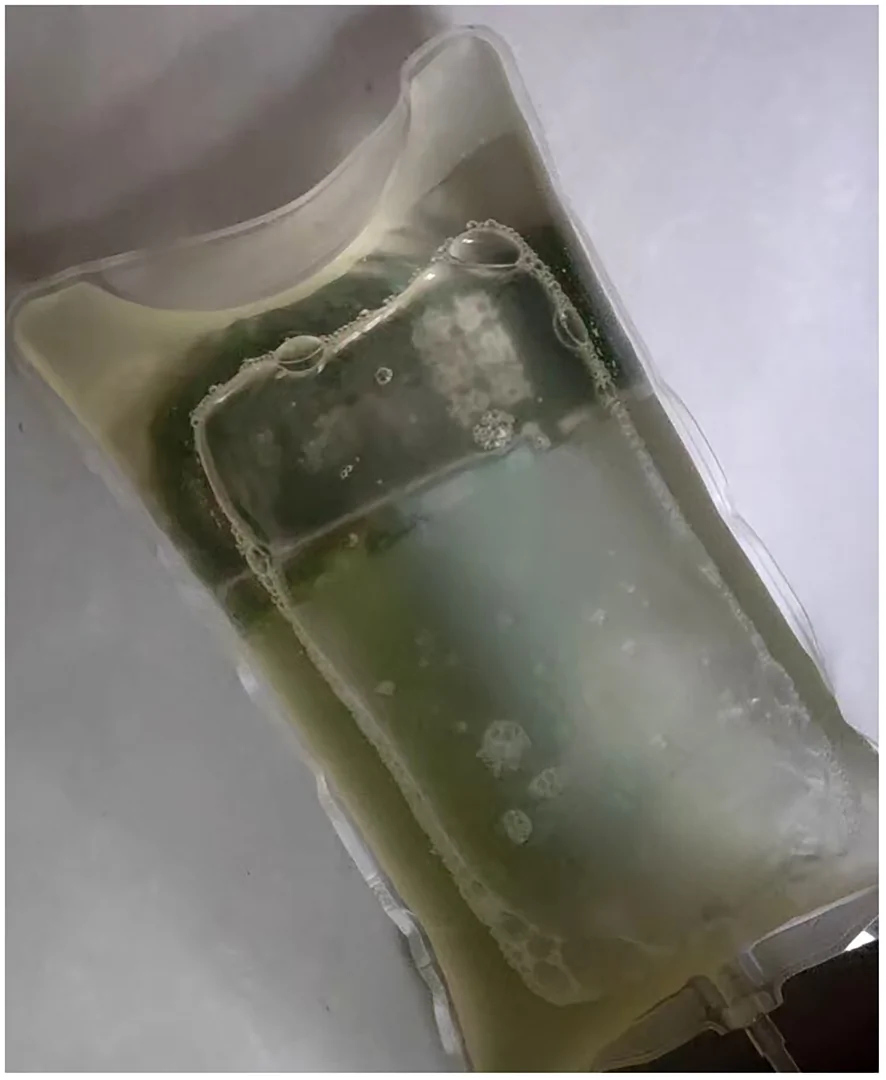
First occurrence of peritoneal dialysis turbidity on January 8, 2026.

On January 9, 2026, the patient continued to have turbid peritoneal dialysate without improvement, with no abdominal pain or diarrhea. Physical examination revealed a blood pressure of 158/110 mmHg, height of 172 cm, weight of 75 kg, BMI of 25.35 kg/m², normal nutritional status, and unremarkable findings on cardiovascular, pulmonary, and abdominal examination. The peritoneal dialysis catheter tunnel and exit site were dry with no exudate, and there was no peripheral edema. Repeat peritoneal dialysate analysis showed a total nucleated cell count of 1293 × 10^6^/L, with 92% mononuclear cells and 8% polymorphonuclear cells. Complete blood count showed a white blood cell count of 5.19 × 10^9^/L, neutrophil count of 3.96 × 10^9^/L, hemoglobin of 110 g/L, and C-reactive protein of 4.75 mg/L. Biochemical parameters included albumin of 37.6 g/L, prealbumin of 443 mg/L, urea of 27.17 mmol/L, creatinine of 1558.47 μmol/L, estimated glomerular filtration rate of 2.6 mL/min/1.73m², bicarbonate of 24 mmol/L, potassium of 4.7 mmol/L, and sodium of 141.7 mmol/L. Serological tests for hepatitis B, hepatitis C, syphilis, and HIV were all negative. Abdominal ultrasound showed increased hepatic parenchymal echogenicity, mild splenomegaly, bilateral kidneys consistent with end-stage renal disease, and mild prostatic enlargement (**Table 1**).

**Table 1:** Changes in routine peritoneal dialysate findings.

Time	WBC (10^6/L)	Mononuclear cells (%)	Polymorphonuclear cells (%)
Jan 8, 2026	543	84	16
Jan 9, 2026	1293	92	8
Jan 11, 2026	143	94	6
Jan 13, 2026	82	98	2
Jan 15, 2026	32	96	4
Jan 17, 2026	5	99.9	0.1
Jan 19, 2026	4	99.9	0.1
Jan 22, 2026	939	82	18
Jan 23, 2026	616	65	35
Jan 24, 2026	142	76	24
Jan 26, 2026	79	86	14
Jan 29, 2026	922	61	39
Jan 30, 2026	34	88	12

On January 9, 2026, cefazolin sodium for injection 1 g and ceftazidime for injection 1 g were administered via intraperitoneal infusion once daily (retained for 8 hours at night). During this period, peritoneal dialysate was sent for blood culture, including both anaerobic and aerobic bottles, which were negative. Fungal G test and fungal GM test were also negative. On January 17, repeat peritoneal dialysate analysis showed a nucleated cell count of 5 × 10^6^/L, and the peritoneal dialysate was clear. Due to the possibility of “culture-negative peritonitis,” antimicrobial therapy was continued.

On January 22, 2026, the peritoneal dialysate became turbid again (see **Figure 2A**) while the patient was still receiving antimicrobial therapy. Physical examination showed no abdominal tenderness, rebound tenderness, or muscle guarding. Repeat peritoneal dialysate analysis revealed a clear, slightly turbid fluid with a positive Rivalta test (±), and a total nucleated cell count of 939 × 10^6^/L, with 82% mononuclear cells and 18% polymorphonuclear cells. The peritoneal dialysate was sent for culture again, which returned negative. On January 25, next-generation sequencing (NGS) of the peritoneal dialysate showed no pathogens detected. On January 26, repeat peritoneal dialysate analysis showed a nucleated cell count of 79 × 10^6^/L, with 86% mononuclear cells and 14% polymorphonuclear cells. Since the nucleated cell count had not decreased to 10 × 10^6^/L, the possibility of “culture-negative peritonitis” could not be ruled out. The patient was planned to complete a 21-day antimicrobial therapy course before discharge.

**Figure 2:**
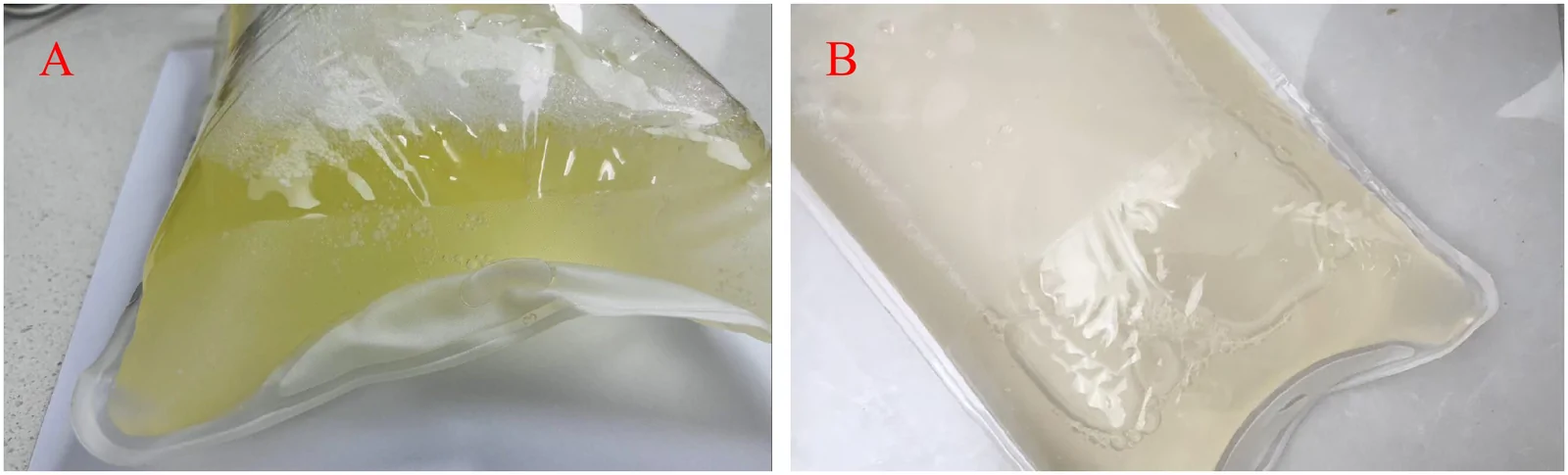
**(A)** Second occurrence of peritoneal dialysis turbidity on January 22, 2026. **(B)** Third occurrence of peritoneal dialysis turbidity on January 29, 2026.

On January 29, 2026, the patient’s peritoneal dialysate became turbid again (see **Figure 2B**). Physical examination showed no abdominal tenderness, rebound tenderness, or muscle guarding. The nucleated cell count in the dialysate was 922 × 10^6^/L. The patient reported that all three episodes of turbid dialysate (on January 8, 2026; January 22, 2026; and January 28, 2026) occurred after self-switching from glucose dialysate to 1.1% amino acid peritoneal dialysate.

The department organized a discussion on complex cases. Upon verification: the patient has been undergoing peritoneal dialysis since November 14, 2022, and the nursing assessment confirmed that the patient’s daily peritoneal dialysis procedures and home management fully comply with standard requirements. There has been no change in the peritoneal dialysis prescription or the manufacturer of the povidone-iodine caps. During the period before and during the current episode, there were no precipitating factors such as respiratory tract infection, unhygienic diet, abdominal pain, or diarrhea. Multiple peritoneal dialysate cultures and NGS tests were all negative. Between January 9 and January 29, the patient did not discontinue or alter the antimicrobial regimen, yet experienced two instances of a significant decrease in nucleated cell count in the dialysate, followed by a marked increase without any adjustment to the antimicrobial treatment. Throughout the hospitalization, the peritoneal dialysate consistently showed an elevation in mononuclear cells, with the ratio of polymorphonuclear to mononuclear cells fluctuating between 0.08 and 0.53, which does not meet the diagnostic criteria for infectious peritonitis.

Based on the patient’s prior prescription records, the physician had exclusively prescribed 1.5% and 2.5% low-calcium peritoneal dialysis solutions, with no other dialysate prescribed. Consequently, at the initial stage of the disease, the clinical team failed to promptly identify the patient’s three instances of unauthorized, temporary use of the 1.1% amino acid peritoneal dialysis solution. It was not until the final episode of peritoneal dialysis effluent turbidity that the patient independently recognized the causal association between the amino acid dialysate and the effluent turbidity. Upon the patient’s disclosure of this critical information to the medical team, a retrospective root-cause analysis was immediately initiated. The chronological sequence of amino acid dialysate administration and effluent turbidity is delineated as follows: On January 8, the patient first self-administered the amino acid peritoneal dialysis solution, and the dialysate became turbid on the same day; on January 9, an intraperitoneal combination therapy regimen was initiated, comprising 1 g of cefazolin sodium injection combined with 1 g of ceftazidime injection (with an 8-hour overnight retention); on January 11, the peritoneal dialysis effluent cleared; on January 22, following the patient’s second unauthorized use of the amino acid dialysate, the effluent became turbid again, during which the original anti-infective regimen was maintained without modification, and the effluent cleared on January 24; on January 29, after the patient’s third unauthorized use of the amino acid dialysate, the effluent became turbid once more, with the antimicrobial regimen remaining uninterrupted and unchanged throughout the period. Based on the clinical course characteristics and therapeutic response, the condition was clinically diagnosed as “peritoneal dialysis-associated chemical peritonitis,” and antimicrobial therapy was immediately discontinued. By January 31, the peritoneal dialysis effluent had spontaneously returned to clarity (**Table 2**).

**Table 2:** Laboratory results and reference intervals.

Time	Project	Subproject	Test result	Reference intervals
Jan 9, 2026	routine blood test	Leukocyte(10^9/L)	5.19	3.5~9.5
		Neutrophi(10^9/L)	3.96	1.1~3.2
		Hemoglobin(g/L)	110	130~175
	Inflammatory markers	C-reactive protein(mg/L)	4.75	0~10
	biochemistry	Albumin(g/L)	37.6	40~55
		Prealbumin(mg/L)	443	200~400
		Urea(mmol/L)	27.17	1.43~7.14
		Creatinine(umol/L)	1558.47	21.5~104
		eGFR (mL/min/1.73m²)	2.6	72~199
		Bicarbonate(mmol/L)	24	22~27
		K^+^(mmol/L)	4.7	3.5~5.3
		Na^+^(mmol/L)	141.7	137~147

During routine follow-up, the patient’s serum albumin consistently exceeded 35 g/L, with a BMI of 25.35 kg/m² and an SGA score of 8, indicating no indication for the use of the amino acid peritoneal dialysis solution. Moreover, the dialysate had been obtained as a transfer from another peritoneal dialysis patient following renal transplantation. Due to this oversight, the medical staff had not been previously informed of the patient’s non-compliant, self-initiated change of dialysate, leading to the initial failure to consider the dialysate itself as the cause of turbidity. Therefore, upon clinical diagnosis of “peritoneal dialysis chemical peritonitis,” both antimicrobial therapy and the use of the amino acid peritoneal dialysis solution were discontinued. Subsequent follow-up through April 2026 revealed no recurrence of dialysate turbidity.

## 3 Discussion

This article reports a case of aseptic peritoneal dialysis-chemical peritonitis induced by the use of 1.1% amino acid peritoneal dialysis solution (Nutrineal). The patient developed dialysate turbidity and a marked increase in mononuclear cells after using the amino acid dialysis solution. However, bacterial and fungal cultures, as well as NGS testing of the peritoneal dialysate, were all negative. The patient did not exhibit abdominal pain, and the routine peritoneal dialysate findings were predominantly elevated mononuclear cells. Symptoms rapidly resolved after discontinuation of the amino acid dialysis solution and switching back to standard glucose dialysis solution, with no recurrence during follow-up, meeting the diagnostic criteria for peritoneal dialysis-chemical peritonitis caused by the amino acid dialysis solution. This case highlights that although amino acid dialysis solution has significant clinical value in improving protein-energy wasting in PD patients, clinicians should remain vigilant for this rare but reversible adverse event. Strict adherence to indications, thorough medical history taking, and comprehensive assessment of potential risk factors for peritoneal dialysis-chemical peritonitis are essential.

The present case is not an isolated phenomenon. Geerse et al. reported eight PD patients who developed sterile peritonitis following exposure to amino-acid-based dialysate, with dialysate leukocyte counts ranging from 150 to 4,900/μL (predominantly mononuclear/macrophages); in all cases, symptoms resolved within 3–7 days after discontinuation of the solution (6). Similarly, a single-center Norwegian experience by Os et al. described comparable findings, indicating that this complication is not limited to a single center (5). In the pediatric population, Kari et al. reported seven children receiving automated peritoneal dialysis (APD) combined with amino-acid-based solution; routine monitoring revealed asymptomatic elevations in peritoneal mononuclear cell counts, presumably attributable to relatively high amino-acid dialysate doses, suggesting that lower doses may be required in children (3). A recent review by Haider et al. reaffirmed the risk of amino-acid-dialysate-associated peritonitis and urged clinical vigilance (2).

The underlying mechanism remains incompletely understood. Current evidence points to a direct effect of amino acids on peritoneal immune cells. Schenk et al. (1994) showed *in vitro* that amino-acid-based dialysate (pH 7.4) suppressed peritoneal macrophage oxidative metabolism and bactericidal capacity more markedly than iso-osmolar glucose-based dialysate, an effect attributed to its high lactate concentration and specific amino acid composition (4). Chan et al. (2003) found that Nutrineal, while preserving human peritoneal mesothelial cell ultrastructure, significantly induced interleukin-6 (IL-6) secretion (P<0.0001), suggesting that amino-acid-based dialysate triggers local cytokine release and subsequent inflammation (8). In the present case, the marked mononuclear-predominant elevation of dialysate nucleated cells implies activation of peritoneal macrophages and mesothelial cells, leading to inflammatory cell recruitment. Contaminants may also play a role: prior reports link icodextrin-induced sterile peritonitis to peptidoglycan contamination, with resolution after withdrawal of affected batches (9). Notably, in the study by Kari et al., leukocyte counts normalized upon discontinuation of amino-acid-based dialysate and rose again upon rechallenge, a reproducible pattern suggesting individual susceptibility (3).

Regarding diagnosis, the International Society for Peritoneal Dialysis (ISPD) defines peritonitis by at least two of the following: (1) abdominal pain and/or cloudy effluent; (2) dialysate leukocyte count >100/μL with >50% polymorphonuclear neutrophils (PMNs); and (3) positive culture (10). The present patient met the first two criteria; however, the PMN proportion was below 50%, cultures remained negative, and symptoms resolved rapidly upon discontinuation of the suspected dialysate—leading to a diagnosis of peritoneal dialysis-chemical peritonitis. Of note, culture-negative peritonitis is not rare in PD patients; de Freitas et al. reported that up to 20% of peritonitis episodes yield negative cultures (11). Thus, when cloudy effluent occurs with negative cultures, peritoneal dialysis-chemical peritonitis should be considered in the differential diagnosis. The following features may help distinguish chemical from infectious peritonitis: peritoneal dialysis-chemical peritonitis typically presents with a lower dialysate leukocyte count (e.g., mean 284 ± 101/mm³ vs. 1,410 ± 973/mm³ in bacterial peritonitis, P<0.05, Touré et al.), a lower neutrophil-to-mononuclear cell ratio (0.67 vs. 4.0), and milder fever and systemic inflammation (11).

Based on the above distinguishing features, when peritoneal dialysis-chemical peritonitis induced by amino-acid-based dialysate is clinically suspected, the following measures should be promptly undertaken: discontinue the amino-acid-based solution and resume conventional glucose-based dialysate; obtain dialysate effluent per standard protocols for bacterial culture and cytology; and closely monitor symptoms and effluent clarity. If marked clinical improvement and progressive clearing occur within 24–72 hours after withdrawal, the diagnosis of peritoneal dialysis-chemical peritonitis is further supported, and antibiotics may be withheld. Conversely, if symptoms persist or worsen, or cultures turn positive, the case should be reassessed and an anti-infective regimen initiated.
